# Isoorientin ameliorates osteoporosis and oxidative stress in postmenopausal rats

**DOI:** 10.1080/13880209.2022.2142614

**Published:** 2022-11-16

**Authors:** Zhilin Cao, Wei Liu, Benjun Bi, Hao Wu, Gong Cheng, Zhongyuan Zhao

**Affiliations:** a Department of Sports Medicine, Yantaishan Hospital, Yantai, China; b Department of Pathophysiology, Binzhou Medical University, Yantai, China; c Department of Hand and Foot Surgery, the Affiliated Hospital of Qingdao University, Qingdao, China; d Department of Articulation surgery, Yantaishan Hospital, Yantai, China

**Keywords:** Biomechanical, 17-β-oestradiol, OPG/RANKL, oestrogen receptor 1

## Abstract

**Context:**

Isoorientin has many biological activities, including antioxidant, anti-inflammatory, antitumor. However, the effect of isoorientin on postmenopausal osteoporosis remains unclear.

**Objective:**

To evaluate the effect of isoorientin on postmenopausal osteoporosis.

**Materials and methods:**

Sprague-Dawley rats were divided into five groups (*n* = 5): sham, model, 17-β-oestradiol (E2, 10 μg/kg/day), low-dose isoorientin (L-Iso, 50 mg/kg), and high-dose isoorientin (H-Iso, 100 mg/kg). The rats were ovariectomized, treated by gavage daily for 12 weeks, and serum and femur samples were collected. Bone mineral density, bone metabolism, and oxidative stress were assessed. H&E staining, immunohistochemistry, and western blotting were employed.

**Results:**

Isoorientin improved the bone mineral density of the lumbar vertebrae (2.01 ± 0.05 g/cm^3^ in H-Iso group vs. 1.74 ± 0.07 g/cm^3^ in model group) and femur (1.46 ± 0.06 g/cm^3^ vs. 1.19 ± 0.03 g/cm^3^), increased the trabecular bone number (1.97 ± 0.03 vs. 1.18 ± 0.13) and thickness (0.27 ± 0.02 vs. 0.16 ± 0.03 mm). Isoorientin decreased the separation degree of trabecular bone, ameliorated bone histomorphology changes, and significantly improved the mechanical properties. Isoorientin diminished MDA (by 60%) and increased SOD (by 49.2%), and GSH-Px (by 159%) activity. Furthermore, osteoprotegerin (OPG), nuclear factor erythroid 2-like 2 (Nrf2), haem oxygenase (HO-1), NAD(P)H quinone dehydrogenase 1(NQO1), and oestrogen receptor 1(ESR1) protein expression increased, while receptor activator of nuclear factor-κB ligand (RANKL) protein expression decreased after treatment.

**Conclusions:**

Isoorientin ameliorates osteoporosis *via* upregulating OPG and Nrf2/ARE signalling, suggesting isoorientin maybe a potential therapeutic drug for PMOP.

## Introduction

Osteoporosis is a disease caused by insufficient bone formation. Undifferentiated stem cells fail to differentiate into osteoblasts after bone resorption, which results in insufficient bone formation. Of the different pathological types of osteoporosis, postmenopausal osteoporosis (PMOP) is the most common, affecting more than 30% of elderly women (Guan et al. 2022). Oestrogen levels, cytokines, and bone metabolism are related to the occurrence of PMOP (Li et al. 2020). The detection of various bone turnover indicators is important for predicting PMOP at the micro level (Kwon et al. 2018). Previous studies have shown that oxidative stress is associated with the occurrence of osteoporosis (Baek et al. 2010; Zhao et al. 2021).

Isoorientin is widely distributed in various plants, including orientin, hawthorn, black buckwheat, *Passiflora caerulea* Linn. (Passifloraceae), and bitter vegetables (Yuan et al. 2016). It is a luteolin 6-C-β-D-glucoside (Lim et al. 2007). Isoorientin has many biological activities, including antioxidant, anti-inflammatory, antitumor, and antibacterial effects (Pei et al. 2019; Fan et al. 2020; Huang et al. 2020; An et al. 2021). However, there is no clear evidence for the effect of isoorientin on PMOP.

OPG/RANKL is an important pathway through which oestrogen participates in osteoclastogenesis and inhibits bone resorption (Zhong et al. 2019). OPG is a transmembrane protein synthesised by osteoblasts. With the differentiation of osteoblasts, OPG can inhibit bone resorption and increase the cortical bone and cancellous bone density, area, and bone strength (Jabbar et al. 2011). RANKL binds to the osteoclast surface receptor RANK; promotes osteoclast formation, differentiation, and maturation; inhibits osteoclast apoptosis; and prolongs osteoclast survival (Kitamura et al. 2013).

Nuclear factor erythroid 2-like 2 (Nrf2) is an important antioxidant protein. When the body produces an oxidative stress response, Nrf2 can rapidly phosphorylate, activate, and translocate to the nucleus, and promote the expression of haem oxygenase (HO-1), superoxide dismutase (SOD), and NAD(P)H quinone dehydrogenase 1 (NQO1) by acting on the downstream ARE protein (Xue et al. 2015). Several cell and animal experiments have confirmed that Nrf2 inhibits oxidative stress-induced osteoblast apoptosis, osteoclast differentiation and maturation, bone microstructure damage, and other pathological changes (Murata et al. 2015; Gambacciani et al. 2018). Few studies have investigated the effects of Nrf2 on the occurrence and development of PMOP. In the current study, we evaluated the effect of isoorientin on rats with PMOP. In addition, we investigated the expression of the Nrf2/ARE signalling pathway in femur tissue to explore the mechanism of isoorientin in PMOP rats and to provide a new theoretical basis for osteoporosis clinical treatment.

## Materials and methods

### PMOP rat model

Twenty-five healthy female Sprague-Dawley (SD) rats (200–220 g) were housed in an SPF animal room at 20–26 °C, relative humidity of 50–60%, and 12-h light/dark cycle. The rats had free access to food and drink. The study was approved by the animal care committee of Yantaishan Hospital (YSLZ2021017), and all experimental procedures were conducted in accordance with the National Institutes of Health (NIH) Guide for the Care and Use of Laboratory Animals.

After one week of adaptive feeding, SD rats were intraperitoneally injected with 1% pentobarbital sodium (40 mg/kg), and the skin was disinfected on the middle section of the spine. A longitudinal incision was made in the median; the skin, subcutaneous, and muscular layers were cut open; and a small incision was made into the abdominal cavity on both sides. Both ovaries were completely removed. After surgery, the rats were injected with penicillin (Sigma-Aldrich, USA) into the abdominal cavity to prevent infection, painkiller (Meloxicam, 2 mg/kg, Merck, USA) was used to relieve postoperative pain for 3 days. All rats were kept under the same conditions and fed in a single cage with standard feed. Five days after the operation, a vaginal epithelial cell smear was observed with a cotton swab, and rats with irregular oestrus for 5 consecutive days were regarded as successful models. One month after the operation, the rat wound healed, and the rats received different treatments.

### Grouping

Rats were randomly divided into five groups (*n* = 5): sham group (all operations were the same as the surgery group, except that the ovaries were not removed), model group, 17-β-oestradiol (E2, 10 μg/kg/day, Sigma, USA), low-dose isoorientin (L-Iso, 50 mg/kg, purity > 98%) obtained from Chengdu Pufei De Biotech Co., Ltd.), and high-dose isoorientin (H-Iso, 100 mg/kg). A continuous gavage was administered daily for 12 weeks. After the last treatment, the rats were fasted for 24 h. The rats were anaesthetised, and their weight was recorded. After the abdominal aortic blood was collected, the rats were sacrificed by cervical dislocation. Serum was separated by centrifugation at 1000 rpm for 10 min and stored at −80 °C for subsequent experiments. The rat femurs were removed, the attached muscles and connective tissues on the femur were peeled off, the left femur was fixed with 10% formaldehyde, and the right femur was frozen at −20 °C until use.

### Bone mineral density (BMD) detection

The L4 lumbar vertebrae and right femur of the rats were removed and scanned using a lunar dual-energy X-ray absorption scanner (GE, USA). The small-object discovery Wi-Fi-scanning mode was used. Scan acquisition time was 4 min and analysis time was 6 min. The area of interest was selected using the instrument, and the centre area was 2.0 × 2.0 mm. BMD was analysed and calculated using software.

### Three-point bending testing

The right femur of each rat was placed on an AG-X (Shimadzu, Japan) series desktop electronic universal testing machine. The span between the two supporting points was 17 mm. The compression point was set at the midpoint of the femur. The loading speed was 2 mm/min, the load-displacement curve was recorded, and the maximum load and elastic modulus were obtained.

### Micro-computed tomography (CT) detection

Before the test, the right femur was thawed at 37 °C, wrapped in tissue paper, and the long axis of the foam ring was fixed and placed in a sample scanning tube. The detection parameters were set as follows: voltage, 80 kV; current, 100 UA; and image matrix, 13% × 2000. A Hiscan XM Micro CT (Hiscan, Suzhou, China), built-in CT-Reconstruct (Hiscan Reconstruct software, V3.0, Suzhou, China), and CT analyser software (Hiscan Reconstruct software, V3.0, Suzhou, China) were used for quantitative parameter analysis. The number of trabecular bones (Tb.N), the degree of trabecular bone separation (Tb.Sp), and trabecular thickness (Tb.Th) were determined.

### Haematoxylin and eosin (H&E) staining and observation

After fixing the femur for 24 h, decalcification was performed using 10% ethylenediaminetetraacetic acid (EDTA) for 30 days. The decalcification solution was replaced every 3 days, and decalcification of the bone tissue was checked during fluid exchange. The femoral tissue was placed in 95% ethanol overnight, gradient dehydrated to 100% ethanol for 1 h per gradient, xylene treated until transparent, embedded in paraffin, and sectioned into 6 μm. Sectional dewaxing with xylene was performed followed by gradient alcohol rehydration, distilled water soaking, haematoxylin (Solarbio, Beijing, China) staining for 20 min, washing in running water back to blue for 10 min, eosin (Solarbio, Beijing, China) staining for 10 min, gradient alcohol dehydration, xylene treatment until transparency, and neutral gum treatment for sealing. Pathological changes in the femoral tissue were observed under an optical microscope (magnification, ×100; Olympus, Japan). Image Pro Plus software (Media Cybernetics, USA) was used to calculate the percentage of the trabecular area (% Tb.Ar = trabecular area/total bone tissue area × 100).

### ELISA

Bone metabolism indices were examined by enzyme-linked immunosorbent assay (ELISA). All indicators were detected according to the manufacturer’s instructions. The optical density (OD) was measured at 450 nm, and the contents of total alkaline phosphatase (ALP), osteocalcin (BGP), cross-linked carboxyl terminal region of type I collagen (CTx-I) (Nanjing Jiancheng Biological Engineering Research Institute, China), and tartrate-resistant acid phosphatase 5 b (TRACP5b, Immunodiagnostic Systems, Gaithersburg, MD, USA) in serum were calculated.

### Detection of oxidative stress markers

The levels of SOD, glutathione peroxidase (GSH-Px), and malondialdehyde (MDA) (Nanjing Jiancheng Biological Engineering Research Institute, China) in serum were detected according to the manufacturer’s instructions.

### Immunohistochemistry

The femoral tissues were routinely sectioned, and the baked flakes were dewaxed with xylene and hydrated sequentially with a gradient ethanol solution. The antigen was repaired in citrate buffer for 20 min, inactivated in 3% hydrogen peroxide solution for 30 min, and blocked with 5% bovine serum albumin (BSA) for 20 min. The primary antibodies rabbit anti-OPG-antibody (1:50, ab73400, Abcam) and anti-RANKL antibody (1:100, sc52950, Santa Cruz Biotechnology, USA) were added dropwise and incubated at 4 °C overnight. After rewarming, the sections were incubated with horseradish peroxidase-labeled goat anti-rabbit IgG and anti-mouse IgG (Abcam, UK) at 37 °C for 1 h. DAB (Solarbio, Beijing, China) was used for colour development, and the sections were lightly counterstained, dehydrated, made transparent, and sealed. Three sections of each tissue were observed under a 100× optical microscope (Olympus, Japan) to analyse the positive cells.

### Western blot assay

The left femur of each group of rats was extracted, and tissue proteins were extracted after grinding. The concentrations of RANKL, OPG, Nrf2, HO-1, NQO1, and ESR1 protein were detected using a BCA Protein Quantitation Kit (Thermo Fisher Scientific, USA). Each group of samples was loaded with 40 μg of protein, separated by sodium dodecyl sulphate-polyacrylamide gel electrophoresis (SDS-PAGE), and transferred to a polyvinylidene fluoride (PVDF) membrane (Millipore, USA). The membrane was blocked with 5% skim milk for 1 h, followed by incubation with primary antibodies anti-OPG antibody (1:1000, sc390518, Santa Cruz Biotechnology, USA), anti-nuclear factor-κB ligand protein (RANKL) (1:1000, sc52950, Santa Cruz Biotechnology, USA), Nrf2 (1:1,000; ab92946, Abcam), anti-HO-1 (1:2,000; ab13243, Abcam), NQO1 (1:500, ab2346, Abcam), and ESR1 (1:1,000; orb216104, Biorbyt, UK) at 4 °C overnight. TBST (TBS, 1 mL/L Tween-20) was used to wash the membrane three times for 5 min each. Horseradish peroxidase-labeled secondary goat anti-mouse (1:5000, sc2005, Santa Cruz Biotechnology, USA) or goat anti-rabbit IgG (1:2000, ab6721, Abcam) was added and incubated for 2 h at room temperature. The membrane was washed with TBST three times for 10 min each, and enhanced chemiluminescence development was performed in a dark room. Protein expression levels were normalised to β-actin and quantified using Image J 1.46 (National Institutes of Health, USA).

### Statistical analysis

IBM SPSS statistical software (version 19.0) was used to perform statistical analysis. All experimental data are expressed as mean ± standard deviation (SD). Data conforms to the normal distribution. One-way analysis of variance (ANOVA) was used to compare groups, and subsequent analysis was performed using the Tukey test. Statistical significance was assumed at *p* < 0.05.

## Results

### Iso treatment ameliorated mineral density of rats

**Figure 1.**
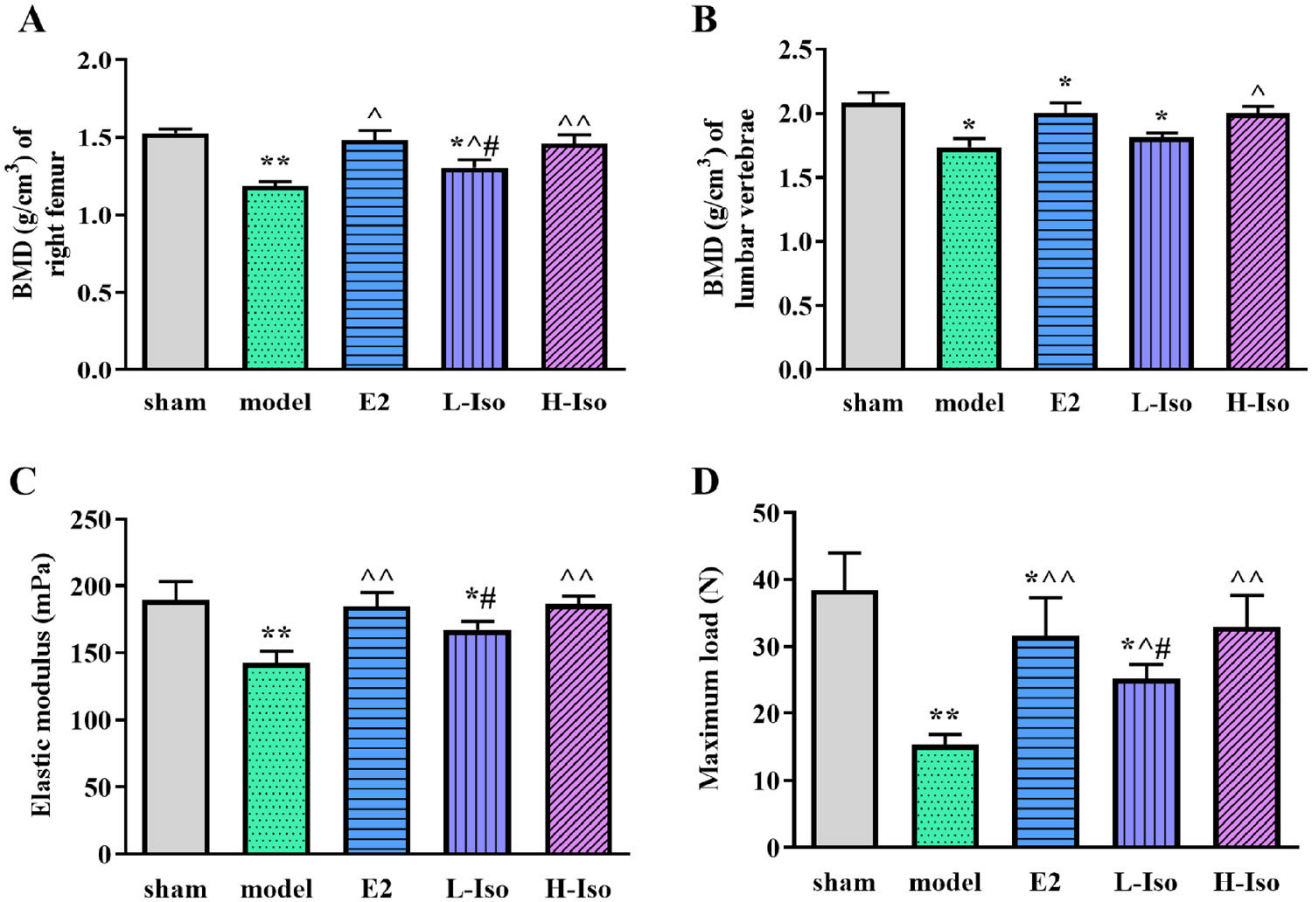
Iso treatment ameliorates bone mineral density and biomechanical indexes of rats. (*n* = 5) A: BMD of right femur; B: BMD of lumbar vertebrae; C: Elastic medulus; D: Maximum load. *p < 0.05, **p < 0.01 compared with the sham group; ^*p* < 0.05, ^^*p* < 0.01 compared with the model group; ^#^*p* < 0.05 compared with the E2 group.

Three months after modelling, the BMD of the rats in each group was measured. Except for the sham group, the BMD of the rats in the other groups was significantly decreased (*p* < 0.05), which proved that the PMOP model was successfully established. After drug intervention, compared with the model group, the BMD values of E2 and Iso groups were significantly increased, and the difference was statistically significant (*p* < 0.05). The BMD value of the H-Iso group was significantly higher than that of the L-Iso group (*p* < 0.05). Furthermore, the BMD value of L-Iso group in femur was significantly lower than that of the E2 group. However, no significant difference was observed between the L-Iso and E2 groups in lumbar vertebrae (Figure 1(A,B), *p* > 0.05).

### Iso treatment ameliorated bone biomechanical

The results of the three-point bending test showed that compared with the sham group, the maximum load and elastic modulus of the femur in the model group were significantly decreased (*p* < 0.05). After drug intervention, compared to the model group, the maximum load and elastic modulus of the femur in the E2 and Iso groups increased (*p* < 0.05), and there was no significant difference between the H-Iso and E2 groups (Figure 1(C,D), *p* > 0.05).

### Iso treatment reversed trabecular structural damage in femur tissue

**Figure 2.**
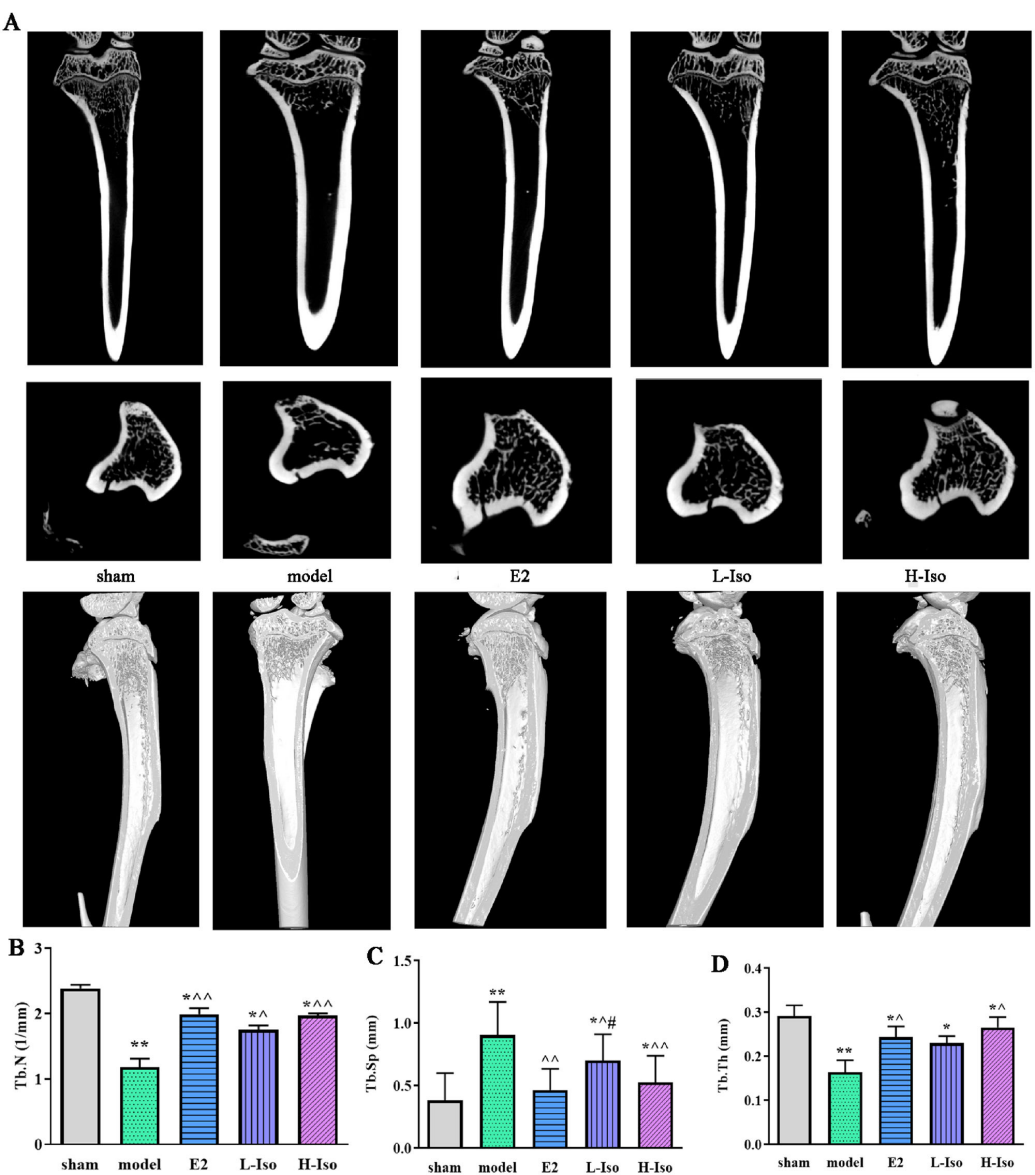
Iso treatment ameliorates trabecular structural damage. (*n* = 5) A. Femur microstructure was investigated with the micro-CT. B: Tb.N; C: Tb.Sp; D: Tb.Th.

Compared to the sham group, the micro-CT results of the model group showed that the Tb.N and Tb.Th decreased. At the same time, Tb.Sp significantly increased. Compared with the model group, E2 and isoorientin treatment improved the Tb.N and Tb.Th, and reduced Tb.Sp. The bone microstructure significantly improved. The Tb.N, Tb.Th, and Tb.Sp in the H-Iso group were significantly different from those in the model group (*p* < 0.05), but were not significantly different from those in the E2 group (*p* > 0.05). Furthermore, the effect of L-Iso was lower than that in E2 group; however, there was no obviously difference (Figure 2, *p* > 0.05).

### Iso treatment reversed trabecular structural damage in femur tissue

**Figure 3.**
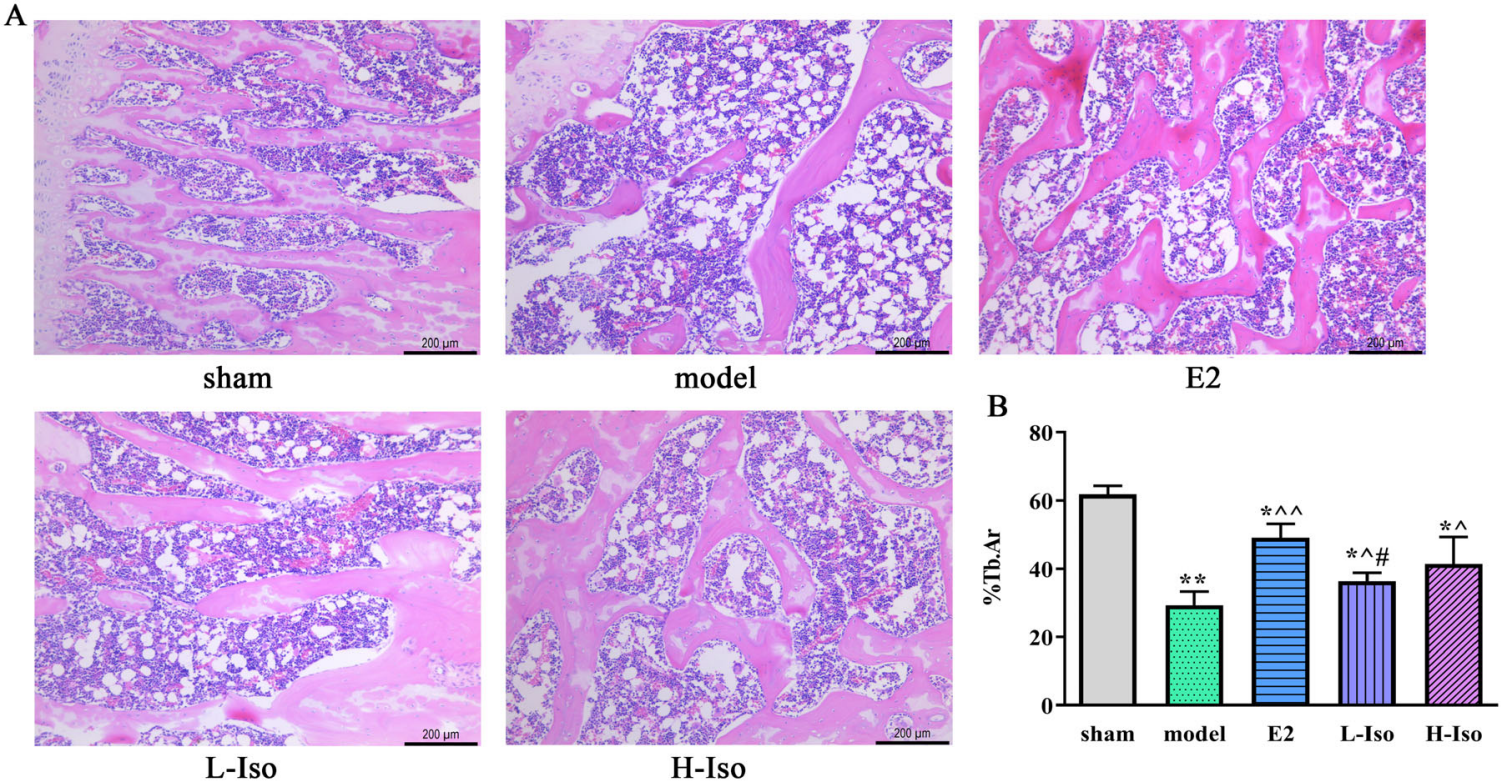
Iso treatment ameliorates morphological changes of femur. (*n* = 5) A: Morphological changes of femur in rats of each group was investigated by H&E staining. (magnification ×100, Scale bar: 200 μm); B: the percentage of trabecular area (% Tb. Ar). *p < 0.05, **p < 0.01 compared with the sham group; ^*p* < 0.05, ^^*p* < 0.01 compared with the model group; #*p* < 0.05 compared with the E2 group.

The H&E staining showed that bone structure appeared normal in the sham group, the trabecular bone was uniform in thickness and complete in structure, the connection between trabeculae was good, and the morphology of adipocytes and osteocytes was clear (Figure 3(A)). In the model group, the structure of the cancellous bone was changed, some bone trabeculae were obviously slender or broken, and the connection between them was incomplete; vacuolar adipocytes increased, which was consistent with the bone morphological damage, indicating that the model was successfully established. The Tb.Ar was increased in the E2, L-Iso, and H-Iso groups than that in model group (*p* < 0.05). Nevertheless, there was no obvious difference between the E2 and L-Iso groups (Figure 3(B), *p* > 0.05).

### Effect of iso treatment on bone turnover markers in the serum

**Figure 4.**
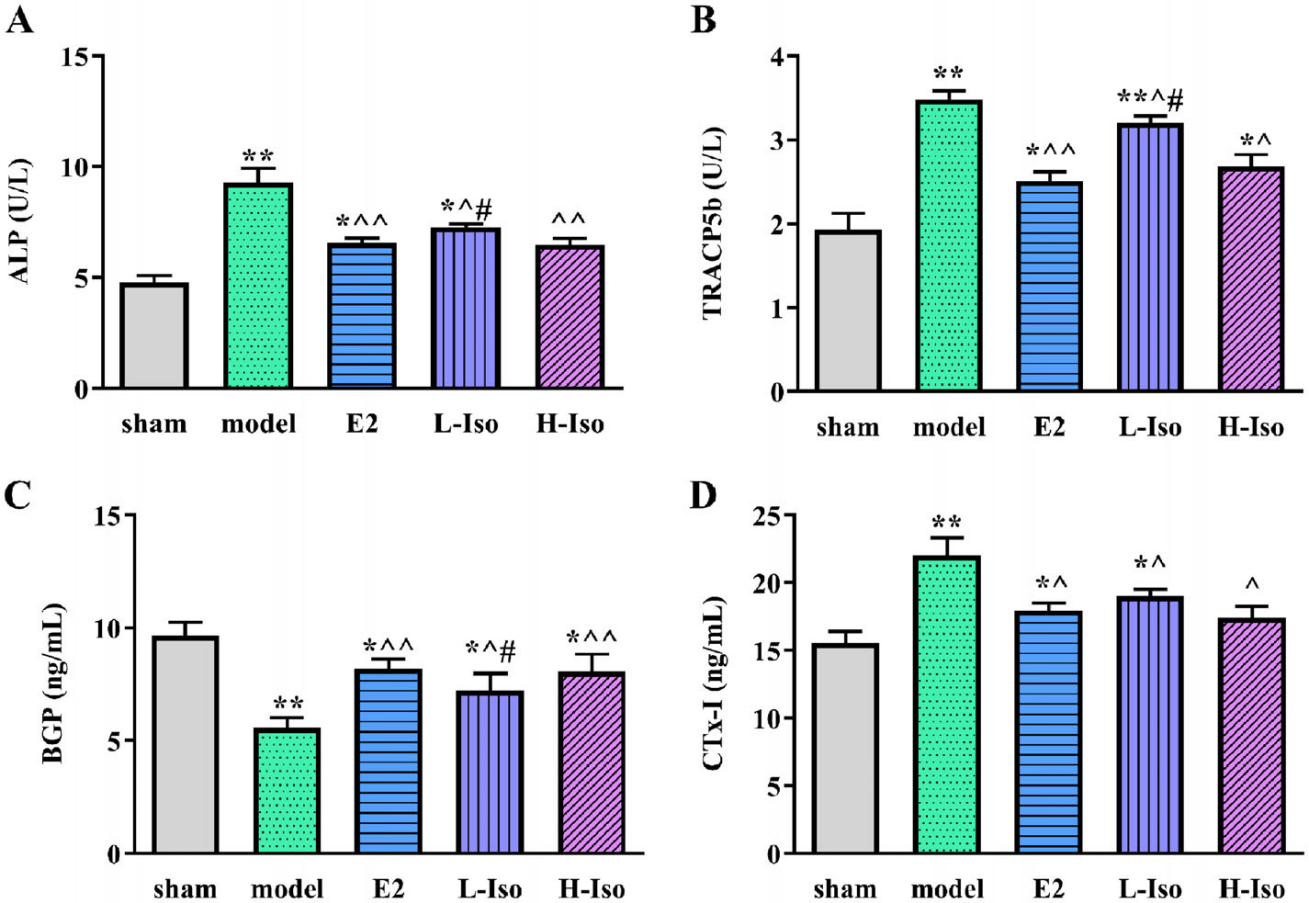
Effect of Iso treatment on bone turnover markers in serum. (*n* = 5) A: ALP; B: TRACP5b; C: BGP; D: CTx-1. *p < 0.05, **p < 0.01 compared with the sham group; ^*p* < 0.05, ^^*p* < 0.01 compared with the model group; ^#^*p* < 0.05 compared with the E2 group.

Compared to the sham group, the BGP concentration in the model group was decreased. Compared with the model group, the serum BGP concentrations of the L-Iso, H-Iso, and E2 groups increased, but there was no significant difference between the H-Iso and sham groups. In contrast, the serum ALP, TRACP5b, and CTx-I levels in the model group were higher than those in the sham group, and the drug treatment groups were decreased (*p* < 0.05). There was no significant difference in ALP and CTx-I levels between the H-Iso and sham groups (Figure 4, *p* > 0.05).

### Effect of iso treatment on oxidative stress markers in the serum

**Figure 5.**
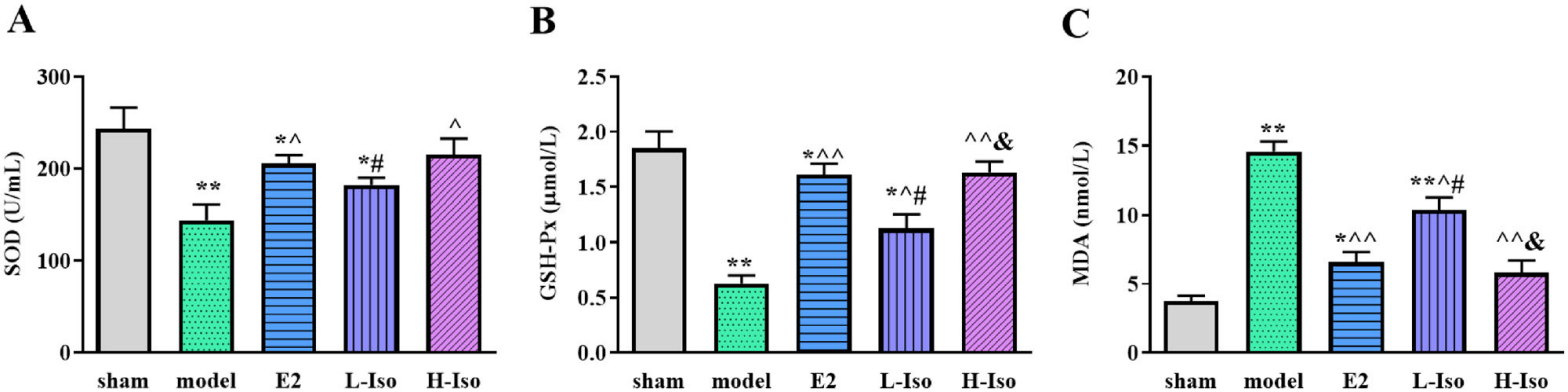
Effect of Iso treatment on oxidative stress index in serum. (*n* = 5) A: SOD; B: GSH-Px; C: MDA. *p < 0.05, **p < 0.01 compared with the sham group; ^*p* < 0.05, ^^*p* < 0.01 compared with the model group; ^#^*p* < 0.05, ^##^*p* < 0.01 compared with the E2 group; ^&^*p* < 0.05 compared with L-Iso group.

The serum SOD and GSH-Px levels in the model group were significantly lower than those in the sham group (*p* < 0.05), and the serum levels in the E2, L-Iso, and H-Iso groups were significantly higher than those in the model group (*p* < 0.05), with the highest level in the H-Iso group. In addition, the serum MDA level in the model group was significantly higher than that in the sham group (*p* < 0.05). After treatment, serum MDA levels in the E2, L-Iso, and H-Iso groups were significantly lower than those in the model group (Figure 5(A–C), *p* < 0.05). The effect of L-Iso group was weaker than those in E2 and H-Iso groups.

### Effect of iso treatment on the expression of RANKL and OPG protein in the femur

**Figure 6.**
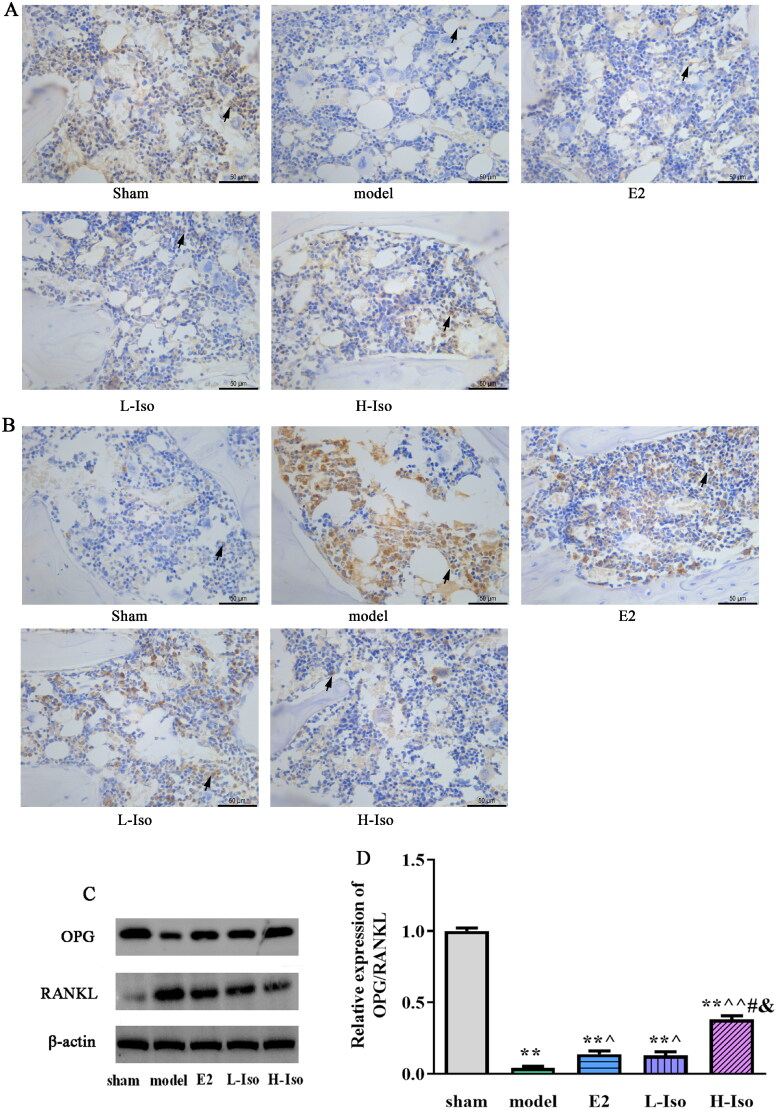
Effect of Iso treatment on the expression of OPG and RANKL protein. (magnification ×400, Scale bar: 50 μm) (*n* = 5) A: Immunohistochemical detection of OPG protein; B: Immunohistochemical detection of RANKL protein; C: Western Blot assay detected the protein expression; D: quantification of OPG protein; E: quantification of RANKL protein. Arrows indicate positive cells. *p < 0.05, **p < 0.01 compared with the sham group; ^*p* < 0.05, ^^*p* < 0.01 compared with the model group; ^#^*p* < 0.05, ^##^*p* < 0.01 compared with the E2 group; ^&^*p* < 0.05 compared with L-Iso group.

The relative number of IHC-positive cells in each group was analysed (Figure 6(A–B)). The OPG level in the model group was significantly lower than that in the sham group, whereas the RANKL level was higher than that in the model group (*p* < 0.05). The relative OPG-positive cell numbers in the E2 and L-Iso groups were significantly higher than those in the model group and were the highest was in the H-Iso group. In contrast, RANKL was significantly decreased in the E2 and L-Iso groups, and it was the lowest in the H-Iso group.

The expression levels of RANKL and OPG were determined using western blotting. The results are presented in Figure 6(C), and the changes were consistent with the IHC assay results. Furthermore, the ratio of OPG/RANKL markedly decreased in model group, while E2 and Iso treatment rescued the decreasing trend. The effect of H-Iso treatment was more significant than that of E2 or L-Iso treatment (*p* < 0.05), and there was no significantly difference between E2 and L-Iso groups (*p* > 0.05).

### Effect of iso treatment on the expression of Nrf2, HO-1, NQO1 and ESR1 protein

**Figure 7.**
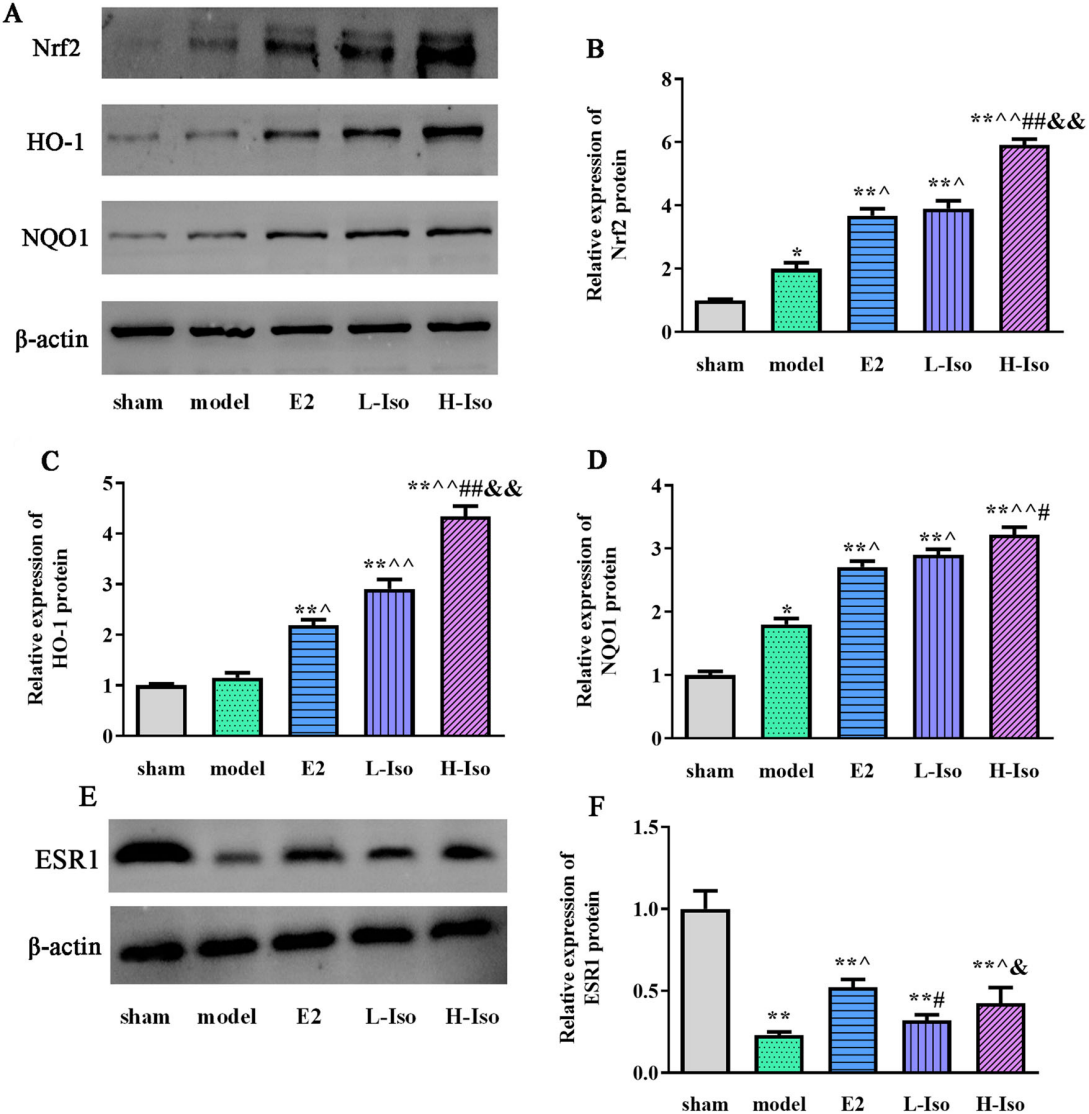
Effect of Iso treatment on the expression of Nrf2, HO-1, NQO1, and ESR1 protein. (*n* = 5) A: Western blot assay detected the protein expression; B: quantification of Nrf2 protein; C: quantification of HO-1 protein; D: quantification of NQO1 protein; E: Western blot assay detected the ESR1protein expression; F: quantification of ESR1 protein. *p < 0.05, **p < 0.01 compared with the sham group; ^*p* < 0.05, ^^*p* < 0.01 compared with the model group; ^#^*p* < 0.05, ^##^*p* < 0.01 compared with the E2 group; ^&^*p* < 0.05, ^&&^*p* < 0.01 compared with L-Iso group.

Western blot analysis showed that the expression of Nrf2, HO-1, and NQO1 proteins in the model group was also higher than that in the sham group (*p* < 0.05) (Figure 7(A–D)). However, the expression of Nrf2, HO-1, and NQO1 in the E2, L-Iso, and H-Iso groups was significantly higher than that in the sham and model groups (*p* < 0.01). In addition, the expression of ESR1 (Figure 7(E), F) in the model group was significantly lower than that in the sham group (*p* < 0.05). The expression of ESR1 in the E2, L-Iso, and H-Iso groups was significantly higher than that in the model group (*p* < 0.05), Furthermore, the expression of ESR1 in the L-Iso group was lower than that in the E2 group (*p* < 0.05).

## Discussion

The current study used micro-CT analysis to present that isoorientin ameliorated bone loss in ovariectomy-induced OP rats. Isoorientin ameliorated bone histomorphology changes, and significantly improved the mechanical properties. Isoorientin diminished MDA, increased superoxide dismutase SOD and GSH-Px activity. The mechanism maybe related to upregulate OPG and Nrf2/ARE signalling. Previous study has shown that isoorientin exert cytoprotective effects on cells *in vitro* (Kuriya et al. 2019). To our knowledge, this is the first study that evaluates the effects of isoorientin on PMOP *in vivo*.

The decrease in oestrogen levels is considered the main mechanism of PMOP. However, osteoporosis is not significantly improved by oestrogen supplementation (Gambacciani et al. 2018). Oestrogen replacement therapy can increase the risk of venous thromboembolism, stroke, and coronary artery disease (Levin et al. 2018). Studies have found that oxidative stress and inflammatory cytokines are involved in the occurrence and development of PMOP (Bonaccorsi et al. 2018). The preventive and therapeutic effects of oestrogen on osteoporosis may be related to its systemic antioxidant and anti-inflammatory effects. The decrease in oestrogen secretion and increase in reactive oxygen species are important reasons for PMOP (Hamidi et al. 2012). The results showed that the levels of SOD and GSH-Px in the serum of postmenopausal osteoporosis rats were significantly decreased, and the levels of MDA were significantly increased, indicating that oxidative stress is involved in PMOP. In the process of occurrence and development, isoorientin can significantly diminish the content of MDA and the metabolite of lipid peroxidation and increase the activities of SOD and GSH-Px. These results showed that isoorientin could improve osteoporosis by inhibiting oxidative stress. Furthermore, oestrogen treatment can also reduce the level of oxidative stress.

Biomechanical properties include the structural and material mechanics of the bone tissue. Common indices include maximum load and elastic modulus. Bone biomechanics is a reliable index for evaluating the effects of drugs on bone quality (Zamani et al. 2018). After 12 weeks of isoorientin treatment, the biomechanical properties of the femoral structure of ovariectomized rats significantly improved, and the maximum load and elastic modulus increased. This indicates that the level of oestrogen in the body decreases after ovariectomy, which increases bone loss and fragility. To a certain extent, isoorientin can improve the biomechanical properties of the femoral structure of ovariectomized rats, increase bone strength, and improve bone stiffness and resistance to degeneration and fracture. In addition, isoorientin also improved the BMD of the lumbar spine and femur, increased the number of trabecular bones and trabecular thickness, decreased the separation degree of trabecular bone, and ameliorated bone histomorphology changes.

Bone metabolism mainly involves osteoclast-mediated bone resorption and osteoblast-mediated bone formation. Bone metabolism can reflect changes in bone turnover, metabolism, and remodelling (Hou et al. 2020). Osteoblasts secrete BGP. Changes in serum BGP levels reflect osteoblasts activity. TRACP-5b reflects the number and functional activity of osteoclasts (Yang et al. 2018). The levels of ALP, TRACP-5b, and CTx-I in the model control group increased, while the level of BGP decreased, indicating that after ovariectomy, the activities of osteoclasts and osteoblasts increased, and the rate of bone turnover increased, leading to increased bone loss. After medication intervention, compared with the model control group, the serum ALP, TRACP-5b, and CTx-I levels of the oestradiol and isoorientin groups were significantly reduced, and the level of BGP was decreased, suggesting that oestradiol and isoorientin treatment can affect osteoclast and osteoblast activities and improve bone metabolism. A previous study investigated the effective components of *Acer palmatum cv. Atropurpureum* (Sapindaceae) leaves, including isoorientin, which has health-promoting effects that help prevent osteoporosis by inhibiting osteoclastogenesis and facilitating osteoblastogenesis (Kuriya et al. 2019). Our study also indicated that isoorientin treatment inhibited osteoclastogenesis and enhanced osteoblastogenesis.

OPG prevents RANKL and RANK interactions by binding to RANKL in adjacent osteoclasts, thereby inhibiting osteoclast formation, differentiation, survival, activation, and induction of osteoclast apoptosis (Wada et al. 2006). The OPG/RANKL ratio is a crucial indicator of osteoblast and osteoclast-related differentiation (Yeom et al. 2021). Our results indicated that protein expression levels of OPG/RANKL significantly decreased in the model group compared with the sham group, whereas E2 and isoorientin groups showed significant increased compared with the model group. Isoorientin increased osteoblast differentiation and reduce bone resorption *via* an increase in the OPG/RANKL ratio.

A previous study indicated that isoorientin upregulated and activated Nrf2 and protected against oxidative damage. Isoorientin induces an increase in the level of NQO1 (Lim et al. 2007). However, the efficacy of isoorientin in postmenopausal osteoporosis remains unclear. In this study, we investigated the expression of Nrf2, HO-1, and NQO1. Compared to the sham group, the expression levels of Nrf2, HO-1, and NQO1 in the femoral tissue of the model group increased slightly, which may be related to the body’s own defense and protection. Compared with the model group, the expression levels of Nrf2, HO-1, and NQO1 increased significantly after intervention with oestrogen and isoorientin, indicating that isoorientin may induce the expression of antioxidant proteins by activating the Nrf2 signal transduction pathway. Isoorientin improved the oxidative stress response of PMOP rats to protect the bone tissue. A previous study indicated that oestradiol reversed the effects of oxidative stress in dermal fibroblasts, probably through the increased activity of ARE/Nrf2 (Darawsha et al. 2021). However, there was no clear evidence of a direct effect of the oestrogen receptor and Nrf2/ARE signalling. In our study, the expression levels of Nrf2, HO-1, and NQO1 were increased compared to those in the model group after E2 treatment, indicating that E2 could ameliorate oxidative stress after PMOP. However, the effect of E2 on Nrf2/ARE signalling was lower than that of Iso. ESR1 is the main oestrogen receptor subtype in bone tissue, and its deletion eliminates the therapeutic effect of oestrogen in the PMOP animal model (Streicher et al. 2017; Xia et al. 2021). We detected changes in ESR1 expression in the different groups. The results indicated that E2 treatment improved the levels of ESR1. However, Iso treatment also ameliorated the levels of ESR1, but the effect was lower than that of E2 treatment.

The findings in this report are subject to at least three limitations. First, the current study does not fully explain the anti-PMOP effects of isoorientin were based on promoting bone formation or inhibiting bone resorption. Second, the impact of oxidative stress on bone mass also not clear. Third, like most studies of PMOP, we do not address the question of OPG/RANKL signalling and Nrf2/ARE pathway relationships. Whilst this study did not confirm the detailed mechanism, it did partially substantiate the anti-PMOP effect of isoorientin. In the future study, the content of bone metabolic index in femur tissue should be determined, the impact of isoorientin anti-oxidative stress on osteoblast also need to investigate. Owing to the side effects of oestrogen treatment alone, the focus of future research should be to explore the synergistic effect of drugs in the prevention and treatment of PMOP.

## Conclusions

This article provides a valuable insight into how isoorientin can reduce oxidative stress, regulate bone metabolism, improve the pathological structure of bone tissue, and play an anti-osteoporotic role in postmenopausal osteoporosis rats. In addition, isoorientin ameliorated bone formation and bone resorption balance *via* OPG/RANKL ratio. Our study indicates that the mechanism of oxidative stress is related to the Nrf2/ARE pathway.
